# Longitudinal Trajectories of Cholesterol from Midlife through Late Life according to Apolipoprotein E Allele Status

**DOI:** 10.3390/ijerph111010663

**Published:** 2014-10-16

**Authors:** Brian Downer, Steven Estus, Yuriko Katsumata, David W. Fardo

**Affiliations:** 1 Sealy Center on Aging, University of Texas Medical Branch, 301 University Blvd., Galveston, TX 77555, USA; 2 Department of Physiology, College of Medicine, University of Kentucky, 138 Leader Avenue, Lexington, KY 40506, USA; E-Mail: steve.estus@uky.edu; 3 Sanders-Brown Center on Aging, University of Kentucky, 101 Sanders-Brown Building, 800 S. Limestone Street, Lexington, KY 40536, USA; 4 Department of Biostatistics, College of Public Health, University of Kentucky, Suite 205, 725 Rose Street, Lexington, KY 40536, USA; E-Mails: katsumata.yuriko@uky.edu (Y.K.); david.fardo@uky.edu (D.W.F)

**Keywords:** cholesterol, life span, aging, Apolipoprotein E

## Abstract

*Background:* Previous research indicates that total cholesterol levels increase with age during young adulthood and middle age and decline with age later in life. This is attributed to changes in diet, body composition, medication use, physical activity, and hormone levels. In the current study we utilized data from the Framingham Heart Study Original Cohort to determine if variations in apolipoprotein E (*APOE*), a gene involved in regulating cholesterol homeostasis, influence trajectories of total cholesterol, HDL cholesterol, and total: HDL cholesterol ratio from midlife through late life. *Methods:* Cholesterol trajectories from midlife through late life were modeled using generalized additive mixed models and mixed-effects regression models. *Results*: *APOE* e2+ subjects had lower total cholesterol levels, higher HDL cholesterol levels, and lower total: HDL cholesterol ratios from midlife to late life compared to *APOE* e3 and *APOE* e4+ subjects. Statistically significant differences in life span cholesterol trajectories according to gender and use of cholesterol-lowering medications were also detected. *Conclusion:* The findings from this research provide evidence that variations in *APOE* modify trajectories of serum cholesterol from midlife to late life. In order to efficiently modify cholesterol through the life span, it is important to take into account *APOE* allele status.

## 1. Introduction

There is a negative perception associated with cholesterol among the general public due to the frequently observed relationship between high cholesterol and adverse health events, such as heart attack and stroke [1]. This perception that cholesterol only has a negative impact on health is somewhat misguided because cholesterol is an essential molecule for healthy functioning of the human body. The liver is the primary source of cholesterol for the body [2], but animal-based food products such as meat, eggs, and cheese are also significant sources of cholesterol [3]. Cholesterol is necessary for maintaining the structural integrity of cell membranes [4] and is a precursor for hormones like testosterone and estrogen and vitamin D, as well as bile salts, which aid in digestion [5]. Cholesterol is transported in the bloodstream by combining with specialized proteins to form lipoproteins. Low density lipoproteins (LDL) and very-low density lipoproteins (VLDL) are more buoyant because they contain more cholesterol in relation to protein. LDL transports cholesterol from the liver through the bloodstream to be used by cells throughout the body [6], and VLDL transports triglycerides and cholesterol to cells to be used for energy [7]. The low-density lipoprotein receptor (LDLR) is responsible for regulating the absorption of cholesterol and triglycerides into the cell [8]. Once VLDL has transported the triglycerides to a cell, it is converted to LDL [9]. High density lipoproteins (HDL) contain more protein in relation to cholesterol and as a result travel more efficiently through the blood stream than LDL and VLDL. HDL cholesterol is commonly referred to as “good cholesterol” because of its role in reverse cholesterol transport in which excess cholesterol is carried back to the liver where it is broken down and excreted from the body by being converted into bile [10,11]. Total cholesterol is the sum of HDL, LDL, and VLDL cholesterol. The ratio of total cholesterol to HDL cholesterol is a commonly used measure of cholesterol in clinical settings because this measure has been found to be more accurate in predicting coronary heart disease than either total cholesterol levels or LDL cholesterol levels [12,13,14]. However, the accuracy of this ratio is questionable at the extreme ends of the total and LDL cholesterol distributions [15]. The National Cholesterol Education Program recommends that for adults, total cholesterol levels should not exceed 200 mg/dL, LDL cholesterol levels should not exceed 100 mg/dL; HDL cholesterol levels should be at least 40 mg/dL for men and 50 mg/dL for women [16]. Based on data from the National Health and Nutrition Examination Survey, the percentage of adults between the ages of 40 and 74 with high LDL cholesterol levels has declined from 59% between 1976–1980 to 27% between 2007–2010 [17]. This decline is due largely to the increasing trend in the number of Americans who have been prescribed cholesterol-lowering medications during the same time period [17]. Maintaining healthy levels of cholesterol is still a significant public health concern because adults with high LDL cholesterol levels or low HDL cholesterol levels are at a substantial risk for cardiovascular diseases [18].

### 1.1. Age Related Changes to Cholesterol

Previous research indicates that total cholesterol levels and LDL cholesterol levels increase with age among young and middle age adults and decline with age later in life [19,20,21,22,23,24]. The relationship between age and HDL cholesterol levels is controversial, with some studies supporting a decrease in HDL cholesterol levels with age, while others have reported minimal change and even an increase in HDL cholesterol levels with age [22,23,25]. There are several factors that contribute to the age related changes in cholesterol. First, LDLR activity tends to decrease with age leading to an increase in circulating LDL as less LDL is absorbed by cells [26]. A decrease in the conversion of cholesterol to bile acid with advancing age also contributes to an increase in serum cholesterol with age [27]. The most consistent predictors across studies for an increase in total cholesterol levels among young and middle aged adults are consuming a high fat diet [28,29] and weight gain [20,30]. The decline in total cholesterol levels among older adults has been observed to coincide with dietary changes [24], weight loss, a decrease in body fat, and the use of statins and other cholesterol-lowering medications [19]. Hormonal changes also contribute to the age related changes in cholesterol observed among women. Young and middle-aged women tend to have lower total cholesterol and higher HDL cholesterol compared to men, but these gender differences become less apparent with increasing age [31]. As women age, total cholesterol levels increase and HDL cholesterol levels may decrease due to a decline in estrogen production preceding menopause [32,33]. Estrogen regulates cholesterol biosynthesis [34] and estrogen replacement therapy is an effective approach for maintaining a healthy cholesterol profile for women who are approaching or experiencing menopause [35,36]. Measures of socioeconomic status, health behaviors, and health characteristics are also associated with total cholesterol levels and HDL cholesterol levels. Low education and low income are associated with poor cardiovascular health [37] and higher total cholesterol [37,38]. These same studies report that adults with high educational attainment and high income also have higher HDL cholesterol levels [37,38]. Also, adults who smoke have lower HDL cholesterol levels compared to non-smokers [39]. Finally, hypertensive adults have higher total cholesterol levels and lower HDL cholesterol levels compared to adults without hypertension [40]. 

Previous studies have reported that the effect of age on cholesterol remains significant even after adjusting for changes in diet, physical activity and the use of statins and other lipid lowering medications [21] suggesting that additional factors contribute to the age related changes in cholesterol. Among these potential factors, we explore here variations in apolipoprotein E (*APOE*), a gene involved in regulating cholesterol homeostasis.

### 1.2. Apolipoprotein E and Cholesterol

Several genes encode proteins that are critical to the absorption, transport, and excretion of cholesterol [41,42]. One such gene is *APOE*. *APOE* is located on the long arm of chromosome 19 (19q13.2) and includes three common alleles (e2, e3, and e4) resulting in six distinct genotypes. The allelic frequency of the *APOE* e2, e3, and e4 alleles among Caucasians is approximately 7%, 78%, and 15%, respectively [43]. The apoE protein is one of several proteins that transport cholesterol and triglycerides through the bloodstream as VLDL and is also involved in the conversion of VLDL to LDL cholesterol and the absorption of cholesterol by the small intestines [44]. While there is debate on if *APOE* genotypes effect the rate of cholesterol absorption [45,46] adults with the *APOE* e3/e3 genotype tend to have lower total cholesterol than adults with either the *APOE* e3/e4 or *APOE* e4/e4 genotypes, but higher total cholesterol compared to adults with either the *APOE* e2/e4, e2/e3 or e2/e2 genotypes [47,48,49]. When environmental factors such as high body mass index (BMI), consuming a high calorie diet and high blood sugar levels are also present, adults with the *APOE* e2/e2 genotype are at an increased risk for type III hyperlipidemia [50], a condition characterized by abnormally high bloodstream concentrations of chylomicrons, which are another lipoprotein that transports triglycerides to cells [7]. 

The observed relationship between *APOE* genotypes and serum cholesterol has led to considerable research on the relationship between *APOE* polymorphisms and diseases associated with high cholesterol. The *APOE* e4 allele is associated with an increased risk for cardiovascular diseases [51], including stroke [52], and coronary heart disease [53]. The *APOE* e4 allele is also an established risk factor for dementia, in particular Alzheimer’s disease (AD) [54], whereas as the *APOE* e2 allele is associated with a decreased risk for AD relative to the *APOE* e3 allele [55]. The consistent association between AD and *APOE* has contributed to research on the relationship between serum cholesterol levels and AD risk. Interestingly, the association between serum cholesterol and AD appears to depend upon the age in which cholesterol is measured. High total cholesterol levels during middle age are associated with an increased risk for AD during old age [56], but older adults with AD tend to have lower total cholesterol compared to non-demented older adults [57] and high cholesterol during old age is associated with a decreased risk for AD [58]. Further, Stewart *et al*. [59] observed in the Honolulu-Asia Aging Study that men who developed dementia exhibited a decline in total cholesterol, on average, 15 years before dementia diagnosis and maintained lower total cholesterol levels during this 15 year period than men who did not develop dementia. A plausible hypothesis for apparent age dependent relationship between serum cholesterol and dementia is that there are distinct differences in serum cholesterol levels from midlife through late life according to *APOE* allele status. This justifies research to examine if trajectories of serum cholesterol from midlife through late life differ according to *APOE* allele status. 

The apoE alleles clearly modulate cholesterol homeostasis as indicated by the differences in cholesterol concentrations among adults according to *APOE* genotypes. However, it is not clear how *APOE* polymorphisms influence the longitudinal trajectories of cholesterol from midlife through late life. Accordingly, the purpose of this study is to characterize the longitudinal trajectories of total cholesterol, HDL cholesterol, and total: HDL cholesterol ratio according to *APOE* e2, e3, and e4 allele status utilizing data from the Framingham Heart Study (FHS) Original Cohort.

## 2. Methods

### 2.1. Framingham Heart Study: Original Cohort

The FHS is an ongoing prospective cohort study of residents from Framingham Massachusetts, United States, created with the goal of generating knowledge on the onset and progression of cardiovascular diseases, as well as genetic and environmental risk factors for these diseases. The FHS Original Cohort was initiated in 1948 and includes adults who did not have a cardiovascular disease upon entry into the study [60]. A total of 5079 subjects between the ages of 28 and 74 (mean age 44.2 years) completed a baseline clinical examination between 1948 and 1953. Thirty clinical examinations have been completed since 1948, and 141 subjects (mean age 92, range 88–102) attended the thirtieth clinical exam, which concluded in 2010. During each wave of data collection, subjects received an extensive physical examination, which included non-invasive tests (e.g., body composition, x-ray, and pulmonary function), lab tests (e.g., lipid and hormone levels), and a health history questionnaire to assess health behavior and the onset of any health conditions since the previous wave of data collection. DNA collection from living members of the FHS Original Cohort began in the 1980s and *APOE* genotype data is available for a total of 663 subjects. For the purposes of the current study, subjects were classified as *APOE* e2+ (e2/e2 or e2/e3 genotype), *APOE* e3 (e3/e3 genotype), or *APOE* e4+ (e3/e4 or e4/e4). Subjects with the *APOE* e2/e4 genotype (n = 11) were excluded from the final sample due to the low sample size and conflicting influence that the *APOE* e2 and *APOE* e4 alleles have on cholesterol levels. Because the purpose of this study was to examine cholesterol trajectories beginning in midlife, nine subjects that were not between 30 and 55 years of age during the first clinical examination were excluded from the final sample. Finally, all subjects had two or more repeated measures for total cholesterol; 21 subjects who had fewer than two consecutive repeated measures for HDL cholesterol were excluded from the final sample. The final sample included 596 subjects. 

### 2.2. Measures of Serum Cholesterol

Measures for total cholesterol and HDL cholesterol were collected from subjects of the FHS Original Cohort using standard laboratory procedures [61]. Fasting measures for total cholesterol were collected during twenty clinical examinations (1–11, 13–15, 20, 22, 24–27) and measures of HDL cholesterol were collected during ten clinical examinations (9–11, 15, 20, 22, 24–27). The ratio of total: HDL cholesterol was calculated by dividing the measure of total cholesterol by the measure of HDL cholesterol. The distributions for total: HDL ratios were highly right skewed and were normalized by log transformation. Serum LDL cholesterol was directly measured in the FHS during three clinical examinations (9–11). The Friedewald equation [62] can be used to estimate LDL cholesterol levels based on total cholesterol, HDL cholesterol, and triglyceride levels (LDL = total – HDL – triglycerides/5). This equation, however, should not be used when triglyceride levels are greater than 400 mg/dL [62] and underestimates LDL cholesterol levels when triglyceride levels exceed 200 mg/dL [63]. Triglyceride levels were measured during nine clinical examinations (7–11, 24–27), but for any given clinical examination over 25 percent of subjects included in the final sample did not have a recorded triglyceride measure. Furthermore, over 13 percent of subjects in each clinical examination had triglyceride levels over 200 mg/dL. In light of these limitations, LDL cholesterol levels were not included in this study. 

### 2.3. Statistical Analysis

The trajectories of total cholesterol levels, HDL cholesterol levels, and total: HDL cholesterol ratios from midlife through late life according to *APOE* allele status were modeled using generalized additive mixed models [64] (GAMM) implemented using the gamm4 package [65] in R version 3.1 GAMM are semi-parametric models that utilize a data-driven approach to model a non-linear relationship between dependent and independent variables as opposed to restriction to fully parametric polynomial terms. The general form of the GAMM used for this study is:

$$Y_{ij}=X_{i}^{T}\beta +s_{e2}\left(x_{ij}\right)I\left(APOE_{i}=e2+\right)+s_{e3}\left(x_{ij}\right)I\left(APOE_{i}=e3\right)+s_{e4}\left(x_{ij}\right)I\left(APOE_{i}=e4+\right)+b_{i}+\epsilon_{ij}$$

where $Y_{ij}$ is the value of cholesterol for the *i*th subject during the *j*th observation given the covariates included in the model (see **Covariates**), and *bi* is a subject-specific random effect that allows for the baseline value of cholesterol to vary for each subject. ***Xi*** is a k-length vector comprising the k time-invariant covariates of subject *i* and ***β*** is a k-length vector of the fixed effects for the corresponding k covariates; *xij* is the age of the *i*th subject during the *j*th observation; *se2*, *se3*, and *se4* are the allele-specific smoothing functions for subjects who were *APOE* e2+, e3, and e4+, respectively, APOEi is the allele status for the ith subject; and I(x) is an indicator function equal to 1 when x is true and 0 otherwise. The term *ϵ_ij_* is the within-subject error term, which is the difference between the observed measure of cholesterol and the expected measure of cholesterol based on the model. The terms *b_i_* and *ϵ_ij_* are assumed to be independent and randomly distributed with mean zero and variances of $\sigma_{b}^{2}$ and $\sigma_{\epsilon }^{2}$ , respectively. The inclusion of three different smoothing functions (*se2*, *se3*, and *se4)* allows for the trajectories of cholesterol to vary according to *APOE* allele status. The smoothing function (thin plate regression splines) models the potentially non-linear trajectory of cholesterol with advancing age. An advantage of the thin plate regression spline function over other smoothing functions is that the number and placement of knots that control the flexibility of the model do not need to be specified [66]. The more knots included in the model, the better the model will fit the data, but this is at the risk of over fitting the data. Concerns of over fitting are addressed by adding a second derivative function on the penalty to the least squares fitting approach [66] providing a tradeoff between model fit and model smoothness [67]. A detailed description of how the thin plate regression spline balances the flexibility and fit of the model has been previously provided [67]. Briefly, the thin plate regression spline estimates the smoothing function *s_i_* by identifying the function $\hat{f}$ that minimizes the equation described below. $\hat{f}$ is the smoothing term that reflects the flexibility of the model

$${\left|y-\text{f}\right|}^{2}+\lambda J_{md}\left(f\right)$$

Where ${\left|y-\text{f}\right|}^{2}$ can be interpreted as the observed data values minus the values predicted from the model, squared; $J_{md}\left(f\right)$ is the penalty function that controls *f*; and λ is the smoothing parameter that controls the balance between the model fit and the smoothness of *f*.

The estimated trajectories of total cholesterol levels, HDL cholesterol levels, and total: HDL cholesterol ratio were plotted along with point-wise 95% confidence intervals constructed by calculating the upper and lower bounds for each predicted measure of total cholesterol levels, HDL cholesterol levels, and total: HDL cholesterol ratio. This allowed for the trajectories of total cholesterol levels, HDL cholesterol levels and total: HDL ratio according to *APOE* allele status to be visually examined. The plots of the estimated trajectories for the adjusted models were obtained by predicting the average values of serum cholesterol based on age and the values of the covariates included in the model. These trajectories represent the average trajectory of serum cholesterol according to *APOE* allele status controlling for the effects of the covariates. The degree to which the trajectories of cholesterol departed from linearity is reflected by the effective degrees of freedom (EDF) of the smoothing parameter (λ) [68]. The EDF provides a measure of a model’s flexibility and a smooth term with *x* EDF can be interpreted as an *x* degree polynomial term. An EDF of 1 indicates that the trajectory is linear and increasing values of EDF indicate greater degrees of non-linearity. Including the EDF values, aids in the interpretation of the estimated trajectories by providing a value for the smoothing function of the trajectory. Also, the potential effects of covariates on the estimated trajectories can be assessed based on the changes to the EDF values as covariates are added to the models. 

**Figure 1:**
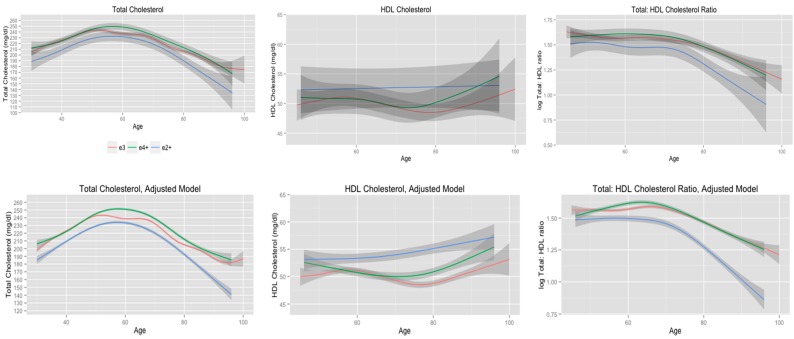
Cholesterol trajectories stratified according to *APOE* e2+, e3, and e4+ allele status.

Notes: *APOE* e4+ includes subjects with e3/e4 or e4/e4 genotypes; e3 includes subjects with e3/e3 genotype; and e2+ includes subjects with e2/e2 or e2/e3 genotypes. Unadjusted cholesterol trajectories (row one) only included *APOE* allele status. Adjusted cholesterol trajectories (row two) controlled for the effects of age gender, educational attainment, smoking status, systolic and diastolic blood pressure, blood sugar, body mass index, restricted diet, and use of cholesterol-lowering medications. Unadjusted trajectories n = 596; adjusted trajectories n = 555. Abbreviations: HDL = high density lipoprotein; APOE = apolipoprotein E.

**Figure 2:**
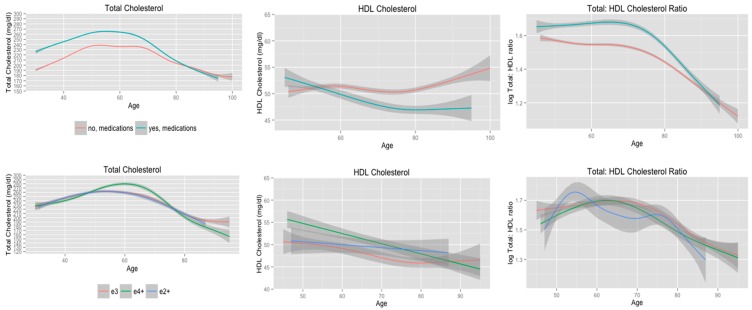
Cholesterol trajectories stratified according to history of using cholesterol-lowering medications, and *APOE* allele status adjusting for age of first using cholesterol-lowering medications.

Notes: *APOE* e4+ includes subjects with e3/e4 or e4/e4 genotypes; e3 includes subjects with e3/e3 genotype; and e2+ includes subjects with e2/e2 or e2/e3 genotypes. Cholesterol trajectories stratified by use of cholesterol-lowering medications (row one) controlled for the effects of age, gender, educational attainment, smoking status, systolic and diastolic blood pressure, blood sugar, restricted diet, body mass index, and *APOE* allele status (n = 555). Cholesterol trajectories stratified according to *APOE* allele status (row two) controlled for the effects of age, gender, educational attainment, smoking status, systolic and diastolic blood pressure, blood sugar, restricted diet, body mass index, and age of first using cholesterol-lowering medications. These trajectories only included subjects who had a history of using cholesterol-lowering medications (n = 102). Abbreviations: HDL = high density lipoprotein; APOE = apolipoprotein E.

**Figure 3:**
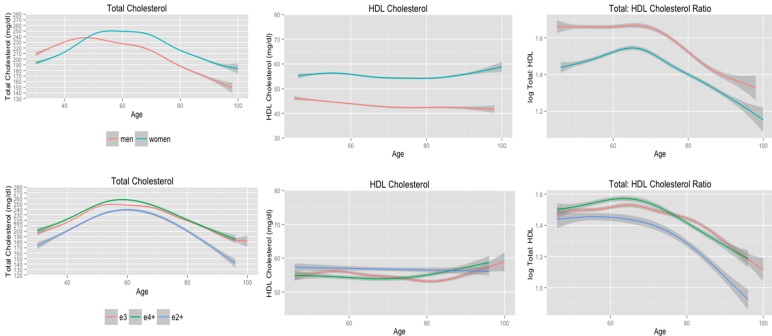
Cholesterol trajectories stratified according to gender, and *APOE* allele status adjusting for age of menopause, use of supplemental estrogen, and cause of menses cessation.

Notes: *APOE* e4+ includes subjects with e3/e4 or e4/e4 genotypes; e3 includes subjects with e3/e3 genotype; and e2+ includes subjects with e2/e2 or e2/e3 genotypes. Cholesterol trajectories stratified according to gender (row one) controlled for the effects of age, educational attainment, smoking status, systolic and diastolic blood pressure, blood sugar, use of cholesterol-lowering medications, restricted diet, body mass index, and *APOE* allele status (n = 555). Adjusted model controlled for the effects of age, educational attainment, smoking status, systolic and diastolic blood pressure, blood sugar, body mass index, restricted diet, *APOE* allele status, age of menopause, use of supplemental estrogen, and cause of cessation of menses (n = 335). Abbreviations: HDL = high density lipoprotein; APOE = apolipoprotein E.

**Figure 4:**
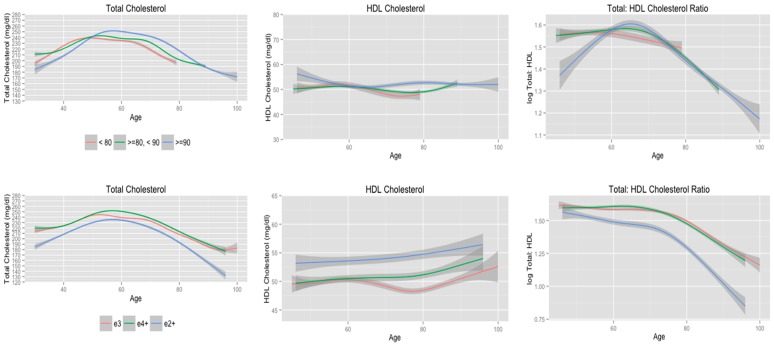
Cholesterol trajectories stratified according to longevity, and *APOE* allele status for subjects who lived beyond 80 years of age.

Notes: *APOE* e4+ includes subjects with e3/e4 or e4/e4 genotypes; e3 includes subjects with e3/e3 genotype; and e2+ includes subjects with e2/e2 or e2/e3 genotypes. All models controlled for the effects of age, gender, educational attainment, smoking status, systolic and diastolic blood pressure, blood sugar, restricted diet, body mass index, and use of cholesterol-lowering medications. Longevity models (row 1) also controlled for the effects of *APOE* allele status Cholesterol trajectories according to longevity (n = 555); cholesterol trajectories according to *APOE* allele status (n = 430); Abbreviations: HDL = high density lipoprotein; APOE = apolipoprotein E.

GAMM allow for between group comparisons to be made based on the 95% confidence intervals of each measure of serum cholesterol, but a challenge when using this method is assessing statistically significant differences in the estimated trajectories of serum cholesterol according to *APOE* allele status or other variable of interest. Since the EDF of the smooth term can be interpreted as an *x* degree polynomial, we used the EDF values obtained from the GAMM as estimates for polynomial terms in mixed-effects regression models [69]. This allowed us to produce evidence for statistically significant differences for the trajectories and aided in the interpretation of the estimated trajectories of serum cholesterol levels presented in the Figure 1, Figure 2, Figure 3 and Figure 4.

### 2.4. Covariates

Data for several potential confounding variables were collected during each clinical examination using standard laboratory procedures and a medical history interview. An overview of the covariates included in the present analysis and the years in which they were collected is provided in Table 1. Covariates were selected according to those included in previous studies that examined longitudinal changes in serum cholesterol levels [20,21]. Also, certain covariates that have been identified as having an effect on serum cholesterol levels were included even if the covariate had not been shown to be associated with *APOE* allele status. Age during the baseline clinical examination was included as a covariate to differentiate between-subject from within-subject variability for the change in serum cholesterol with age. Educational attainment was initially recorded according to the following categories: (1) none, (2) fourth grade or less, (3) fifth, sixth or seventh grade, (4) completed grade school, (5) some high school, (6) graduated high school, (7) some college, (8) college graduate or(9) post graduate. For the purposes of this study, educational attainment was dichotomized according to receiving a high school (HS) degree (≤HS or >HS). From clinical examination 7 through 21, subjects were asked if they were using cholesterol-lowering drugs, and it was not until clinical examination 22 that subjects were asked which type of medication they used (resins, niacin, fibrates, or statins). Since specific cholesterol-lowering medications were not assessed until the 22nd clinical examination, subjects were dichotomized as having reported ever using a cholesterol-lowering medication (yes/no). The age in which subjects first started using cholesterol-lowering medications was included as a covariate in a subsequent analysis to account for differences in the timing in which subjects started taking cholesterol-lowering medications. Also, an analysis limited to only women that controlled for the use of supplemental estrogen and age of menopause was also conducted. During the 1st through 14th clinical examinations, women were asked the age in which menses had stopped for one year or more and the cause of cessation (natural, surgical, or other). The average age of menopause was 47.5 years (SD = 6.0, range = 23–57 years), but significant differences in the average age of menopause according to cause of cessation were detected (natural (n = 242) 49.9 years, SD = 3.4; surgery (n = 108) 42.3 years, SD = 7.1; other (n = 3) 41.7 years, SD = 7.6; F-value = 92.9, *p* < 0.01). Therefore, the cause of cessation of menses was included as a covariate in this analysis. The use of supplemental estrogen (oral, patch, or cream) was assessed during clinical exams 17–27. Subjects were dichotomized as having reported ever using supplemental estrogen (yes/no). Subjects were asked during clinical exams 2, 4, 7–15, and 17 if they were consuming a diet meant to control high cholesterol, diabetes, hypertension, weight, or other low calorie diet. Subjects were dichotomized as reported ever consuming a restricted diet (yes/no). Systolic blood pressure, diastolic blood pressure, height (inches) and weight (pounds) were collected during each clinical examination. Smoking status was assessed during clinical exams 4–27. Blood sugar levels were measured during clinical exams 1, 2, 4, 6, 8–23, 26 and 27. Body mass index (BMI) was calculated as (weight/height^2^) × 703. Smoking status [70], blood pressure [71], BMI [72] and blood sugar [73] have been observed to change with age. Therefore, we included measures for these covariates during the 1st clinical examination (representing midlife) and 15th clinical examination (representing late life). This accounted for potential changes in these variables from midlife to late life and limited the number of covariates included in each model since these variables were measured during almost all clinical examinations. The 1st clinical examination represented midlife because only subjects between the ages of 30–55 during this examination were included in the final sample. Smoking status during the 4th clinical examination was included as a covariate since smoking was not assessed during the 1st through 3rd clinical examinations. Only 12 subjects (2.0% of final sample) did not attend the 4th clinical examination. The average age of the subjects who did attend the 4th clinical examination was 44.3 years (range = 35–61). The 15th clinical examination was selected for late life because the average age of the final sample was 66.3 years (range = 58–83 years). These cut offs for midlife and late life are consistent with those used in previous studies [74,75]. A total of 570 subjects (95.6% of final sample) attended the 15th clinical examination. Covariate values for the 14th or 16th clinical examination were used for subjects who did not attend the 15th clinical examination.

## 3. Results

### 3.1. Description of Framingham Heart Study: Original Cohort

The descriptive characteristics of subjects of included in the final sample are provided in Table 2. The final sample included a total of 596 subjects, of which 61 were *APOE* e2+ (n = 4 e2/e2; n = 57 e2/e3), 406 were *APOE* e3, and 129 were *APOE* e4+ (n = 5 e4/e4; n = 124 e3/e4). The final sample was predominantly female and the majority of subjects had a high school degree or less. There were no statistically significant differences in *APOE* allele status according to any of the characteristics listed in Table 2. Compared to women, men were more likely to be current smokers during midlife (men = 149 (65.1%), women = 135 (38.8%), *χ^2^* = 44.4, *p* < 0.01) and late life (men = 79 (33.3%), women = 74 (21.3%), *χ^2^* = 56.6, *p* <0.01), had high average BMI during midlife (men = 25.7 kg/m^2^, women = 24.7 kg/m^2^, t-value = −3.4, *p* < 0.01) and late life (men = 27.2 kg/m^2^,women = 26.5 kg/m^2^, t-value = –2.2, *p* = 0.03), higher average blood sugar during late life, but not midlife, (men = 95.7 mg/dL, women = 91.7, t-value = –2.0, *p* < 0.05), higher average systolic blood pressure during midlife, but not late life, (men = 131.5 mmHg, women = 126.3 mmHg, t-value = −4.0, *p* < 0.01), and higher average diastolic blood pressure during midlife (men = 82.6 mmHg, women = 80.3 mmHg, t-value = −2.6, *p* = 0.01 ) and late life (men = 78.1 mmHg, women = 75.5 mmHg, t-value = –3.1, *p* = 0.02).

### 3.2. Total Cholesterol Stratified by APOE Allele Status

The unadjusted and adjusted trajectories of total cholesterol, HDL cholesterol, and total: HDL cholesterol ratio, according to *APOE* allele status, are presented in Figure 1. Solid lines represent the mean measures of serum cholesterol according to *APOE* status and the shaded regions represent the point-wise 95% confidence intervals for each estimated measure. The degree of non-linearity for the trajectories as reflected by the estimated EDF of the smoothing term, and the model fit by adjusted R2, which is interpreted as the proportion of total variance explained by the model with an adjustment for model complexity, are presented in Table 3. According to visual inspection, there were little to no differences in the unadjusted mean measures of total cholesterol, HDL cholesterol, and total: HDL cholesterol ratio as indicated by the overlap of the 95% confidence intervals. Inclusion of covariates to the models noticeably reduced the variability in estimated cholesterol trajectories as indicated by the 95% confidence intervals for each measure of cholesterol; however, trajectories of cholesterol did not substantially change once covariates were added to the models as indicated by the consistent EDF values of the smooth terms. The dramatic reduction in the 95% confidence intervals for the adjusted models compared to the unadjusted models suggests that several factors account for the variability in serum cholesterol levels. Including covariates reduces the error variance of the model, which reduces the variability of the estimates of serum cholesterol.

Total cholesterol trajectories for *APOE* e2+, e3, and e4+ subjects all followed similar non-linear trends based on the EDF values (Table 3. Significant differences according to *APOE* allele status were detected after controlling for the effects of age, gender, educational attainment, smoking status, BMI, systolic and diastolic blood pressure, blood sugar levels, restricted diet, and use of cholesterol-lowering medications (Figure 1. Subjects who were *APOE* e2+ maintained consistently lower total cholesterol from midlife through late life compared to *APOE* e3 and *APOE* e4+ subjects. *APOE* e3 and *APOE* e4+ subjects had similar trajectories for total cholesterol until 50 years of age, at which point *APOE* e3 subjects displayed a decline in total cholesterol while total cholesterol in *APOE* e4+ subjects continued to increase until 60 years of age. The greatest difference in total cholesterol between *APOE* e3 and *APOE* e4+ subjects was at 60 years of age and the differences in total cholesterol between these two groups became less apparent with advancing age. A mixed-effects regression model that included linear through 7th order polynomial terms for age detected statistically significant differences in the interaction terms from *APOE* allele status and quadratic (F-value = 4.7, *p* < 0.01) and quartic(F-value = 8.2, *p* < 0.01) terms, while the cubic term approached statistical significance(F-value = 2.9, *p* = 0.06).

**Table 1:** Assessment of total cholesterol, HDL cholesterol, and selected covariates from the Framingham Heart Study Original Cohort.

Measure	Measure Collected																										
Age	*	*	*	*	*	*	*	*	*	*	*	*	*	*	*	*	*	*	*	*	*	*	*	*	*	*	*
Weight	*	*	*	*	*	*	*	*	*	*	*	*	*	*	*	*	*	*	*	*	*	*	*	*	*	*	*
Height	*	*	*	*	*	*	*	*	*	*	*	*	*	*	*	*	*	*	*	*	*	*	*	*	*	*	*
Diastolic BP	*	*	*	*	*	*	*	*	*	*	*	*	*	*	*	*	*	*	*	*	*	*	*	*	*	*	*
Systolic BP	*	*	*	*	*	*	*	*	*	*	*	*	*	*	*	*	*	*	*	*	*	*	*	*	*	*	*
Smoking				*	*	*	*	*	*	*	*	*	*	*	*	*	*	*	*	*	*	*	*	*	*	*	*
Diet		*		*			*	*	*	*	*	*	*	*	*		*										
Estrogen use																	*	*	*	*	*	*	*	*	*	*	*
Menopause cause	*	*	*	*	*	*	*	*	*	*	*	*	*	*													
Menopause	*	*	*	*	*	*	*	*	*	*	*	*	*	*													
Cholesterol medications							*	*	*	*	*	*	*	*	*	*	*	*	*	*	*	*	*	*	*	*	*
Sex	*																										
Education	*																										
Blood sugar	*	*		*		*		*	*	*	*	*	*	*	*	*	*	*	*	*	*	*	*			*	*
HDL cholesterol									*	*	*				*					*		*		*	*	*	*
Total cholesterol	*	*	*	*	*	*	*	*	*	*	*		*	*	*					*		*		*	*	*	*
**Clinical Exam**	**1**	**2**	**3**	**4**	**5**	**6**	**7**	**8**	**9**	**10**	**11**	**12**	**13**	**14**	**15**	**16**	**17**	**18**	**19**	**20**	**21**	**22**	**23**	**24**	**25**	**26**	**27**
**Years**	**1948**	**1950**	**1952**	**1954**	**1956**	**1968**	**1960**	**1962**	**1964**	**1966**	**1968**	**1971**	**1972**	**1975**	**1977**	**1979**	**1981**	**1983**	**1985**	**1986**	**1988**	**1990**	**1992**	**1995**	**1997**	**1999**	**2001**

Notes: Subjects were evaluated every 2 years beginning in 1948. Clinical examination 27 was completed in 2003.

**Table 2:** Demographic characteristics of final sample (*n*=596).

Characteristic	Value
Avg. age, midlife (range, SD)	38.3 (30.0–55.0, 5.5)
Gender, *n* (%)	
Men Women	242 (40.6) 354 (59.4)
Educational attainment	
≤High school >High school	387 (66.2) 198 (33.8)
Smoking midlife, *n* (%), *n* = 577	
Non-smoker Former Current	282 (48.9) 11 (1.9) 284 (49.2)
Smoking, late life, *n* (%), *n* = 585	
Non-smoker Former Current	231 (39.5) 201 (34.3) 153 (26.1)
Use of cholesterol-lowering medications, *n* (%)	
No Yes	487 (81.7) 109 (18.3)
* Avg. age cholesterol medication use (SD, range)	69.7 (45.0–88.0, 11.4)
Avg. midlife blood sugar, mg/dL (SD, range), *n* = 590	79.4 (13.5, 51.0–173.0)
Avg. late life blood sugar, mg/dL (SD, range), *n* = 584	93.3 (24.2, 52.0–326.0)
Avg. midlife systolic blood pressure, mmHg (SD, range)	128.4 (16.0, 90.0–195.0)
Avg. late life systolic blood pressure, mmHg (SD, range), *n* = 588	135.9 (19.5, 92.0–212)
Avg. midlife diastolic blood pressure, mmHg (SD, range)	81.3 (10.4, 50.0–125.0)
Avg. late life diastolic blood pressure, mmHg (SD, range), *n* = 588	76.6 (9.8, 50.0–104.0)
Avg. midlife body mass index, Kg/M^2^ (SD, range)	25.1 (3.8, 15.4–41.2)
Avg. late life body mass index, Kg/M^2^ (SD, range), *n* = 588	26.7 (4.3, 16.7–43.7
** Supplemental estrogen, *n* (%)	
No Yes	315 (89.0) 39 (11.0)
Dietary restriction, *n* (%)	
No Yes	345 (57.9) 251 (42.1)
*APOE* allele status, *n* (%)	
e2+ e3 e4+	61 (10.2) 406 (68.1) 129 (21.6)
Avg. number of cholesterol measures (range, SD)	
Total cholesterol HDL cholesterol	14.4 (4.0–19.0, 2.5) 5.1 (2.0–10.0, 1.8)

Notes: Midlife and late life were the 1st and 15th examinations, respectively; * The average age in which subjects first reported using cholesterol-lowering medications; ** Supplemental estrogen use was assessed in only women.

**Table 3:** EDF and adjusted R2 for trajectories of total, HDL, and total: HDL cholesterol ratio stratified according to *APOE* allele status.

Model	Total Cholesterol		HDL Cholesterol		Total: HDL Cholesterol	
	EDF	Adjusted R^2^	EDF	Adjusted R^2^	EDF	Adjusted R^2^
Unadjusted model		0.12		0.01		0.06
e2+	4.9		1.0		3.8	
e3	7.7		3.6		4.4	
e4+	5.7		2.8		3.3	
Adjusted model		0.22		0.19		0.20
e2+	4.9		1.0		3.7	
e3	7.5		3.7		4.7	
e4+	5.6		2.9		3.3	

Notes: Unadjusted model only included *APOE* allele status (*n* = 596); Adjusted model controlled for the effects of age, gender, educational attainment, smoking status, systolic and diastolic blood pressure, blood sugar, restricted diet, body mass index, and use of cholesterol-lowering medications (*n* = 555); Abbreviations: EDF = effective degrees of freedom; HDL = high density lipoprotein; APOE = apolipoprotein E.

In general, *APOE* e2+ subjects had higher HDL cholesterol levels compared to *APOE* e3 and *APOE* e4+ subjects (Figure 1). *APOE* e2+ subjects displayed a gradual increase in HDL cholesterol from midlife through late life, whereas HDL cholesterol declined until approximately 75 years of age, which was followed by an increase in HDL cholesterol, for *APOE* e3 and *APOE* e4+ subjects. The greatest difference in HDL cholesterol between *APOE* e2+, e3, and e4+ subjects was observed between 75 and 80 years of age. A mixed-effects regression model that included linear, quadratic, and cubic terms for age did not detect any statistically significant differences in these polynomial terms according to *APOE* allele status. However, the linear (F-value = 6.6, *p* = 0.01) and cubic (F-value = 21.1, *p* < 0.01) main effect polynomial terms were statistically significant.

All subjects maintained consistent total: HDL cholesterol ratio until 70 years of age when the trajectories began to decline (Figure 1). *APOE* e2+ subjects maintained significantly lower total: HDL ratios from midlife through late life compared to *APOE* e3 and *APOE* e4+ subjects and these differences increased with advancing age. There were little to no differences in the trajectories of total: HDL cholesterol ratio according to *APOE* allele status. A mixed-effects model that included linear through quartic polynomial terms for age and interactions with *APOE* allele status detected a statistically significant interaction between age and *APOE* allele status (F-value = 3.2, *p* = 0.04). The respective interactions between *APOE* allele status and the other three polynomial terms were not statistically significant. 

### 3.3. Cholesterol Trajectories according to Use of Cholesterol-Lowering Medications

The trajectories of serum cholesterol levels stratified according to use of cholesterol-lowering medications are presented in the first row of Figure 2. In general, subjects who had a history of taking cholesterol-lowering medications had higher serum total cholesterol levels, lower HDL cholesterol levels, and higher total: HDL cholesterol ratios from midlife through late life compared to subjects who did not have a history of taking cholesterol-lowering medications. Subjects who had a history of using cholesterol-lowering medications had similar EDF values for total cholesterol, HDL cholesterol, and total: HDL cholesterol ratio compared to non-medication users (Table 4). Medication users maintained higher total cholesterol from midlife through 80 years of age, but these subjects had a greater decline in total cholesterol following 60 years of age compared to non-medication users. These apparent differences in the trajectories of serum total cholesterol are consistent with the results of a mixed-effects model that included linear through 7th order polynomial terms for age. This model detected statistically significant differences in the *APOE* allele status by linear (F-value = 22.2, *p* < 0.01), quadratic (F-value, *p* = 0.04), and cubic (F-value = 4.7, *p* < 0.03) interaction terms. 

Medication users had higher HDL cholesterol levels during midlife compared to non-medication users. Medication users, however, did have a slight decline in HDL cholesterol until 80 years of age, whereas non-medication users displayed a gradual increase until 60 years of age, which was followed by a slight decline. Both medication users and non-users exhibited an increase in HDL cholesterol levels following 80 years of age. These findings are supported by the results of a mixed-effects regression model that included linear, quadratic, and cubic polynomial terms for age. There were significant linear (F-value = 6.7, *p* = 0.01) and cubic trends (F-value = 21.1, *p* < 0.01) in HDL cholesterol according to age for all subjects, but only the *APOE* allele status by age interaction term was statistically significant (F-value = 7.9, *p* = 0.01). 

Finally, subjects with a history of using cholesterol-lowering medications maintained significantly higher total: HDL cholesterol ratio compared to non-medication users from midlife through late life (row 1, Figure 2). However, medication users did have a greater decline in total: HDL cholesterol ratio following 70 years of age. This is supported by the findings of a mixed-effects regression model that included linear, quadratic, and cubic polynomial terms for age. In this model the age^2^ by *APOE* allele status interaction term was statistically significant (F-value = 7.6, *p* = 0.01).

The second row of Figure 2 presents cholesterol trajectories according to *APOE* allele status adjusting for the effects of age of first using cholesterol-lowering medications. *APOE* e2+, e3, and e4+ subjects had similar trajectories of serum total cholesterol through 50 years of age. Total cholesterol continued to increase among *APOE* e4+ subjects until 60 years of age, whereas total cholesterol began to decline at 50 years of age for *APOE* e3 and *APOE* e2+ subjects. The EDF values for the smooth terms were similar for *APOE* e4+, e3, and e2+ subjects (Table 3). However, a mixed-effects model that included linear through quartic polynomial terms for age detected statistically significant differences in the quadratic (F-value = 3.3, *p* = 0.04) and quartic (F-value = 9.6, *p* < 0.01) polynomial terms according to *APOE* allele status while the cubic interaction term approached statistical significance (F-value = 2.5, *p* = 0.08).

*APOE* e4+ had significantly higher HDL cholesterol levels during midlife compared to *APOE* e3 and *APOE* e2+ subjects (row two, Figure 2). *APOE* e4+ subjects, however, did exhibit the greatest decline in HDL cholesterol with age. According to the EDF values for the smooth terms *APOE* e4+ and *APOE* e2+ subjects displayed linear declines in HDL cholesterol, whereas the trajectory of HDL cholesterol for *APOE* e3 subjects showed a slight quadratic trend (Table 4). There was considerable overlap in the 95% confidence intervals for the estimated values of HDL cholesterol, which suggests there is not a significant difference in the trajectories of HDL cholesterol according to *APOE* allele status. This is supported by the results of a mixed-effects regression model that included linear and quadratic polynomial terms for age interactions with *APOE* allele status. In this model, the linear term for age was statistically significant (F-value = 16.2, *p* < 0.01), but there was not a statistically significant interaction between age and *APOE* allele status. 

There was substantial overlap in the 95% confidence intervals for estimated values of total: HDL cholesterol ratio according to *APOE* e4 allele status (row two, Figure 2). Based on the EDF values for the smooth terms (Table 4), *APOE* e4+ and e3 subjects exhibited cubic trends in total: HDL cholesterol ratio. *APOE* e3 and *APOE* e4+ subjects maintained consistent total: HDL cholesterol ratio until 70 years of age, at which point the trajectories began to decline. The total: HDL cholesterol ratio trajectory for *APOE* e2+ subjects followed a quartic trajectory (Table 4). The ratio of total: HDL cholesterol increased from 45–55 years of age, decreased until 60 years of age, plateaued until 75 years of age, and declined dramatically after 75 years of age. These visibly evident differences in total: HDL cholesterol ratio trajectories according to *APOE* allele status are supported by the results of a mixed-effects regression model that included linear through quartic polynomial terms for age in which the age^4^ by *APOE* allele status interaction was the only statistically significant interaction term(F-value = 3.5, *p* = 0.03).

**Table 4:** EDF and adjusted R2 for trajectories of total, HDL, and total: HDL cholesterol ratio stratified according to use of cholesterol lowering medications, and *APOE* allele status adjusting for age of first using cholesterol lowering medications.

Model	Total Cholesterol		HDL Cholesterol		Total: HDL Cholesterol	
	EDF	Adjusted R^2^	EDF	Adjusted R^2^	EDF	Adjusted R^2^
Use of cholesterol-lowering medications		0.22		0.19		0.16
No	7.2		3.7		4.0	
Yes	5.4		2.5		3.7	
Adjusted model		0.26		0.28		0.20
e2+	3.4		1.0		3.8	
e3	4.6		1.5		3.3	
e4+	4.6		1.0		2.6	

Notes: Model for use of cholesterol-lowering medications controlled for the effects of gender, educational attainment, smoking status, systolic and diastolic blood pressure, blood sugar, restricted diet, body mass index, and *APOE* allele status (*n* = 555). Adjusted model controlled for the effects of age, gender, educational attainment, smoking status, systolic and diastolic blood pressure, blood sugar, restricted diet, body mass index, *APOE* allele status, and age of first using cholesterol-lowering medications (*n* = 102). Abbreviations: EDF = effective degrees of freedom; HDL = high density lipoprotein; APOE = apolipoprotein E.

### 3.4. Cholesterol Trajectories Stratified by Gender, and APOE Allele Status Adjusting for Menopause

Cholesterol trajectories stratified according to gender are presented in the first row of Figure 3. During midlife, women had significantly higher total cholesterol levels compared to men, but women exhibited an increase in total cholesterol until 55 years of age compared to 45 years of age for men. The EDF values were similar for men and women, but women showed a greater decline in serum total cholesterol with age compared to men. These findings are supported by the results from a mixed-effects regression model that included linear through 7th order polynomial terms for age and interactions terms with *APOE* allele status. This model detected statistically significant differences in the linear (F-value = 149.6, *p* < 0.01), quadratic (F-value = 35.5, *p* < 0.01), quartic (F-value = 43.0, *p* < 0.01), and quintic (*i.e.*, age^5^; F-value = 9.3, *p* < 0.01) polynomial terms according to *APOE* allele status.

Both men and women maintained consistent levels of HDL cholesterol (first row, Figure 3; Table 5). Women had significantly higher HDL cholesterol levels from midlife through late life compared to men. The estimated trajectories for HDL cholesterol and the EDF values indicate that there is a between group effect of sex on HDL cholesterol, but that the trajectories do not differ according to sex. A mixed-effects model that included linear, quadratic, and cubic polynomial terms for age along with interactions with *APOE* allele status supports this finding. There were no significant differences in any of the polynomial terms included in this model according to *APOE* allele status. 

Finally, men maintained significantly higher total: HDL cholesterol ratio compared to women from midlife through late life (first row, Figure 3). The EDF values for the smooth terms were very similar for men and women, but there is evidence for significant differences in these trajectories. A mixed-effects model that included linear through quartic polynomial terms for age detected statistically significant differences in the linear interaction term (F-value = 6.6, *p* = 0.01) and the quadratic interaction term approached statistical significance (F-value = 3.6, *p* = 0.06).

**Table 5:** EDF and adjusted R2 for trajectories of total, HDL, and total: HDL cholesterol ratio according to gender, and *APOE* allele status adjusting for age of menopause.

Model	Total Cholesterol		HDL Cholesterol		Total: HDL Cholesterol	
	EDF	Adjusted R^2^	EDF	Adjusted R^2^	EDF	Adjusted R^2^
Gender		0.25		0.19		0.20
Male	6.2		3.0		4.5	
Female	7.3		4.2		4.3	
Adjusted		0.25		0.08		0.15
e2+	5.0		1.0		2.9	
e3	7.1		4.2		4.6	
e4+	5.5		2.6		3.1	

Notes: Model for gender controlled for the effects of educational attainment, smoking history, average systolic and diastolic blood pressure, average blood sugar, restricted diet, average body mass index, use of cholesterol-lowering medications, and *APOE* allele status (*n* = 555). Adjusted model controlled for the effects of educational attainment, smoking, systolic and diastolic blood pressure, blood sugar, restricted diet, body mass index, *APOE* allele status, age of menopause, use of supplemental estrogen, and cause of cessation of menses (*n* = 335). Abbreviations: EDF = effective degrees of freedom; HDL = high density lipoprotein; APOE = apolipoprotein E.

Cholesterol trajectories stratified according to *APOE* allele status among women adjusted for the effects of supplemental estrogen, age of menopause, and cause of menses cessation are presented in the second row of Figure 3. The EDF values for the smooth terms are provided in Table 5. Women who were *APOE* e2+ maintained significantly lower total cholesterol and total: HDL cholesterol ratio from midlife through late life compared to women who were *APOE* e4+ or *APOE* e3. However, mixed-effects models for total cholesterol, which included linear through 7th order polynomial terms for age, and total: HDL cholesterol ratio, which included linear through quartic polynomial terms for age, did not detect statistically significant differences in these trajectories according to *APOE* allele status. All women maintained consistent levels of HDL cholesterol and there were little to no differences in HDL cholesterol according to *APOE* allele status from midlife through late life. These findings are consistent with those from a mixed-effect model that did not detect any statistically significant differences in linear through quartic polynomial terms according to *APOE* allele status. 

### 3.5. Cholesterol Trajectories Stratified according to Longevity

An explanation for the observed decline in total cholesterol levels, increase in HDL cholesterol levels and decline in total: HDL cholesterol ratio is that subjects with healthy cholesterol levels are more likely to reach very old age. The majority of subjects included in the final sample lived beyond 80 years of age (n = 460, 77.2%), which included 133 subjects who lived past 90 years of age. Compared to subjects who did not live beyond 80 years of age, subjects who were 80 years of age or older were more likely to be female (n = 290 (63.0%), n = 64 (47.1%), *χ^2^* = 11.1, *p* < 0.01), and less likely to have been a smoker during midlife (n = 95 (72.5%), n = 189 (42.4%), *χ^2^* = 37.8, *p* < 0.01) and late life (n = 57 (43.8%), n = 96 (21.1%), *χ^2^* = 30.7, *p* < 0.01). 

Since there were statistically significant differences in the characteristics of the final sample according to longevity, a subsequent analysis was conducted in which the trajectories of total cholesterol, HDL cholesterol, and total: HDL cholesterol were stratified according to longevity (age of death < 80 years, ≥80 to <90, and ≥90). These models adjusted for the effects of age, educational attainment, smoking, systolic and diastolic blood pressure, blood sugar, BMI, restricted diet, and use of cholesterol-lowering medications. 

The trajectories for total cholesterol, HDL cholesterol, and total: HDL cholesterol ratio are presented in the first row of Figure 4 and the corresponding EDF values are presented in Table 6. Subjects who lived beyond 90 years of age had the lowest total cholesterol levels during midlife, but the highest levels for total cholesterol during old age. Compared to subjects who did not live beyond 80 years, subjects who lived between 80 and 90 years had similar total cholesterol levels during midlife and slightly higher total cholesterol levels, most notably after 70 years of age. The serum total cholesterol trajectories according to longevity were examined further with a mixed-effects model that included linear through 7th order polynomial terms with age and interactions according to longevity status. This model detected statistically significant interactions between longevity and the linear (F-value = 21.7, *p* < 0.01), quadratic (F-value = 6.3, *p* < 0.01), quartic (F-value = 5.0, *p* = 0.01), and quintic (F-value = 9.2, *p* < 0.01) polynomial terms. 

There were little to no differences in the estimated trajectories of HDL cholesterol according to longevity. This is supported by the findings from a mixed-effects model that included linear, quadratic, and cubic polynomial terms for age and interactions with longevity status. In this model, the cubic polynomial term was statistically significant (F-value = 20.6, *p* < 0.01), but there was not a statistically significant interaction between age^3^ and longevity. 

Finally, subjects who did not live past 80 years had a linear decline in total: HDL cholesterol ratio, whereas total: HDL cholesterol ratio for subjects who lived past 80 and 90 years of age did not begin to decline until approximately 65 years of age. Interestingly, subjects who lived past 90 years of age had low total: HDL cholesterol ratio that increased until approximately 60 years of age. This visibly apparent difference in the trajectory of total: HDL cholesterol ratio is detected in a mixed-effects model that included linear, quadratic, and cubic terms for age in which the interaction term between age^2^ and longevity was statistically significant (F-value = 3.0, *p* = 0.05). 

**Table 6:** EDF and adjusted R2 for trajectories of total, HDL, and total: HDL cholesterol ratio according to longevity, and *APOE* allele status among long-lived subjects.

Model	Total Cholesterol		HDL Cholesterol		Total: HDL Cholesterol	
	EDF	Adjusted R^2^	EDF	Adjusted R^2^	EDF	Adjusted R^2^
Longevity		0.22		0.19		0.20
<80	5.3		3.1		1.0	
≥80 to <90	6.8		3.6		4.0	
≥90	6.7		1.0		3.3	
*APOE* allele status		0.22		0.16		0.16
e2+	5.0		1.0		3.2	
e3	7.3		2.1		3.8	
e4+	5.8		1.0		3.1	

Notes: All models controlled for the effects of age, gender, educational attainment, smoking status, systolic and diastolic blood pressure, blood sugar, restricted diet, body mass index, and use of cholesterol-lowering medications. Longevity models also controlled for the effects of *APOE* allele status; Cholesterol trajectories according to longevity (*n* = 585); cholesterol trajectories according to *APOE* allele status (*n* = 430).

A final analysis limited to the 460 subjects who lived beyond 80 years of age (77% of final sample) was conducted to assess if trajectories of total cholesterol, HDL cholesterol, and total: HDL cholesterol ratio according to *APOE* allele status differed once subjects who did not live beyond 80 years were removed. These models adjusted for the effects of age, educational attainment, smoking history, average systolic and diastolic blood pressure, average blood sugar, average BMI, restricted diet, and use of cholesterol-lowering medications. It should be noted that *APOE* e3 subjects remained in the FHS Original Cohort longer compared to *APOE* e2 and *APOE* e4 subjects as indicated by the five more years of observation for *APOE* e3 subjects (Figure 4). There were minimal differences in the trajectories and EDF values of total cholesterol, HDL cholesterol, and total: HDL cholesterol ratio (second row, Figure 4; Table 6) compared to the trajectories and EDF values that included all subjects in the analysis (second row, Figure 1; Table 3). The findings from the mixed-effects models for these trajectories were also consistent with the models that included the entire sample. The mixed-effects model for total cholesterol detected statistically significant interactions between *APOE* allele status and the quadratic (F-value = 5.1, *p* = 0.01) and quartic (F-value = 6.6, *p* = 0.01) polynomial terms for age. As was observed in the initial analysis, there were no statistically significant interactions between any of the polynomial terms for age and *APOE* allele status in the mixed-effects model for HDL cholesterol. Finally, the mixed-effects model of total: HDL cholesterol for subjects who lived beyond 80 years of age detected a statistically significant interaction between age and *APOE* allele status (F-value = 4.2, *p* = 0.02), whereas the interactions between *APOE* allele status and other polynomial terms (quadratic, cubic, and quartic) were not statistically significant. This is consistent with the results from the mixed-effects model that included the entire final sample. To summarize, these findings provide evidence that the results from the initial analysis are not due to a healthy survivor bias. 

## 4. Discussion

The findings from this study present new evidence for age related changes in total cholesterol, HDL cholesterol, and total: HDL cholesterol ratio according to *APOE* allele status. Results from the GAMMs indicated that *APOE* e2+ subjects maintained lower serum total cholesterol levels, higher HDL cholesterol levels, and lower total: HDL cholesterol ratios from midlife through late life compared to *APOE* e3 and *APOE* e4+ subjects after controlling for the effects of age gender, educational attainment, smoking, use of cholesterol-lowering medications, systolic and diastolic blood pressure, blood sugar, diet restriction, and BMI. Similar findings were observed when subjects who did not live beyond 80 years of age were excluded. Further analysis using mixed-effects regression models detected statistically significant interactions between polynomial terms for age and *APOE* allele status for trajectories of serum total cholesterol. This provides evidence that trajectories of serum total cholesterol from midlife through late life differ according to *APOE* allele status. However, a mixed-effects regression model for HDL cholesterol did not detect any statistically significant interactions between *APOE* allele status and polynomial terms for age. This suggests that there is a between group effect for *APOE* on HDL cholesterol levels, but there are not substantial differences in HDL cholesterol trajectories according to *APOE* allele status. Finally, there was a statistically significant interaction between age and *APOE* allele status in the mixed-effects model for total: HDL cholesterol ratio, but the interactions between *APOE* allele status and quadratic, cubic, and quartic polynomial terms were not statistically significant. These findings, when interpreted with the results from the GAMM, indicate there is a significant between group effect for *APOE* allele status on total: HDL cholesterol trajectories and that *APOE* e2+ subjects have a greater overall decline in total: HDL cholesterol ratio than *APOE* e3 or *APOE* e4+ subjects. 

Prior research has focused on age-related changes to total and HDL cholesterol levels through the lifespan and the present analysis is the first study to our knowledge that has examined trajectories of serum cholesterol from midlife through late life stratified according to *APOE* allele status. In the current study, serum total cholesterol levels increased with age during midlife but declined during old age. These results are consistent with other studies that have examined age related changes in serum total cholesterol [19,20,21,22,24,25]. We observed an increase in HDL cholesterol from midlife through late life, but there were no statistically significant differences in HDL cholesterol trajectories according to *APOE* allele status. This increasing trend in HDL cholesterol with age as been reported previously [25], but other studies have observed consistent levels [22] or a decline [20] in HDL cholesterol with age. 

Previous studies have also assessed sex differences in serum cholesterol levels. The findings from the GAMM and mixed-effects models provide evidence for significant differences in the trajectories of serum cholesterol between men and women. Women had lower serum total cholesterol during midlife, but higher total cholesterol during late life compared to men. Women also had significantly higher HDL cholesterol and total HDL cholesterol and lower total: HDL cholesterol ratio from midlife through late life compared to men. These results are consistent with previous research [31,76]. It should be noted that we chose not to stratify all analyses according to sex because sex differences in the trajectories of serum cholesterol levels according to *APOE* allele status were not the objective of the present analysis. Future research should examine how sex may modify the relationship between *APOE* allele status and trajectories of serum cholesterol levels from midlife through late life.

The decline in total cholesterol with increasing age among older adults observed in the current study may be the result of a survivor effect in which subjects at an increased risk for mortality due to diseases caused by elevated cholesterol die and subjects with healthy cholesterol levels remain in a study into old age [77]. In the current study, subjects who lived beyond 90 years had lower total cholesterol during midlife, but higher total cholesterol during late life, compared to subjects who lived between 80 and 90 years. Subjects who lived between 80 and 90 years had higher total cholesterol levels during late life compared to subjects who did not live past 80 years, but there were little to no differences in midlife total cholesterol levels between these two groups. There is evidence from previous research that a decrease in cholesterol synthesis by the liver and a decline in the amount of dietary cholesterol absorbed by the intestines contributes to the decline in total cholesterol later in life [78,79,80]. A survivor effect may also partially explain the increase in HDL cholesterol after 80 years of age. Subjects who lived beyond 80 years of age were more likely to be female and less likely to smoke during midlife and late life. These characteristics have been associated with higher levels of HDL cholesterol [39] and the overall better health of subjects who lived past 80 years may explain the increase in HDL cholesterol among the oldest-old in the FHS Original Cohort. 

Certain limitations in this study need to be acknowledged. First, we limited the number of covariates included in the adjusted models by using midlife and late life values for potentially important covariates such as smoking status, measures for systolic and diastolic blood pressure and blood sugar, use of cholesterol-lowering medications, and BMI. This approach allowed us to account for changes in these covariates from midlife through late life, but may have oversimplified the relationship between these variables and trajectories of serum cholesterol levels. Furthermore, we controlled for the use of cholesterol-lowering medications by dichotomizing subjects as ever using medications (yes, no). It has been suggested that adding a constant value to observed values for subjects receiving treatment is more appropriate when controlling for treatment effects than including treatment status as a dichotomous covariate [81]. These limitations can be addressed by future research that focuses on how changes in specific health behaviors and health conditions through the life span influence trajectories of serum cholesterol from midlife through late life. A second potential limitation is that the FHS did not provide a definition for what constituted a restricted diet. Clinical trials indicate that diets rich in whole grains, fruits, nuts, and vegetables have beneficial effects on cardiovascular disease risk factors including high cholesterol, diabetes, and hypertension [82]. We were unable to assess the dietary preferences and caloric intake among subjects who reported consuming a restricted diet because the FHS did not introduce a detailed food history questionnaire until the 20th clinical examination. Only 71 subjects (11.9%) included in the final sample were still remaining in the FHS Original Cohort during the 20th examination and controlling for the effects of dietary preferences and caloric intake would have considerably reduced the sample size. Therefore, our findings need to be replicated using other sample populations from studies that have assessed caloric intake throughout the life span since older adults can experience decreased appetite [83] and malnutrition [84], which may contribute to a decline in cholesterol during old age. Another limitation of the present analysis is we did not control for potentially important factors that modify or are related to serum cholesterol levels such as alcohol consumption [85], physical activity [86], cardiovascular diseases [87], and inflammation [88]. Additional research on the effects that these variables, as well as other genetic, biological, lifestyle, and sociocultural factors, have on serum cholesterol levels through the life span is warranted. 

## 5. Conclusions

In summary, this study presents evidence that serum total cholesterol and total: HDL cholesterol ratio trajectories from midlife through late life differ according to *APOE* allele status. Between-group differences in HDL cholesterol according to *APOE* allele status were also detected. 

The findings from this study have important implications to public health. First, we observed that adults who lived past 90 years of age had higher total cholesterol during late life compared to adults who did not live past 80 or 90 years of age. This has potentially important implications for the treatment of high cholesterol with statins and other cholesterol-lowering medications because these findings suggest that higher cholesterol may be associated with longevity. An argument can be made that it may be harmful to prescribe older adults statins or other medications to lower cholesterol based on evidence that low serum cholesterol and a decline in serum cholesterol are associated with increased mortality [89,90]. However, it is not clear if a decline in cholesterol plays a causal role in mortality or this decline is a consequence of changes to systems involved in cholesterol homeostasis, such as liver function, cholesterol metabolism, and appetite, that are negatively affected by biological changes that occur during the period of terminal decline prior to death [91]. Second, the *APOE* e4 allele is associated with an increased risk for several aging related diseases, including AD [54] and cardiovascular diseases [92], whereas the *APOE* e2 allele has been associated with a decreased risk for these diseases [55]. To efficiently modify cholesterol, and as a result, disease risk, it is necessary to take into account *APOE* allele status and how *APOE* allele status influences serum cholesterol levels from midlife through late life. A plausible hypothesis is that the increased risk for cognitive and vascular diseases among older adults who carry one or more *APOE* e4 alleles may be due, in part, to higher total cholesterol and lower HDL cholesterol from midlife through late life, whereas the decreased risk for these diseases associated with the *APOE* e2 allele may be due to the lower total cholesterol and higher HDL cholesterol across the life span. This hypothesis is based on interpreting the findings of the present analysis within the context of findings from previous research. Additional research is necessary to determine if reducing total cholesterol and increasing HDL cholesterol decreases the risk for cognitive and vascular diseases among adults who carry one or more *APOE* e4 alleles.
